# Mr. W. B. Saunders

**Published:** 1905-11

**Authors:** 


Mr. W. B. SAUNDERS, of Philadelphia, one of the largest medical book publishers in this country, died suddenly at Atlantic City on October 1, 1905. He had suffered from a nervous disorder for some months, due to too close attention to his large business interests. Mr. Saunders began the publishing business in 1891, and from the start was recognised as issuing books of superior quality and workmanship.—*Medical Bulletin.*

